# Hypertriglyceridemia and its impact on mitotane monitoring in adrenocortical carcinoma

**DOI:** 10.1530/EDM-23-0014

**Published:** 2023-12-01

**Authors:** Sandra Martens, Bruno Lapauw

**Affiliations:** 1 Ghent University Hospital, Ghent, Belgium; 2 Ghent University, Ghent, Belgium

**Keywords:** Adult, Male, White, Belgium, Adrenal, Endocrine-related cancer, Adrenal, Endocrine-related cancer, Unusual effects of medical treatment, December, 2023

## Abstract

**Summary:**

Mitotane is used for treatment of advanced adrenocortical carcinoma. It is administered when the carcinoma is unresectable, metastasized, or at high-risk of recurrence after resection. In addition, mitotane is considered to have direct adrenolytic effects. Because of its narrow therapeutic–toxic range, therapeutic drug monitoring (TDM) is warranted. In 2020, a left-sided adrenal gland tumor was found (5.8 cm) in a 38-year-old man. Considering the size of this lesion and inability to exclude an adrenocortical carcinoma on imaging, a laparoscopic adrenalectomy was performed. Histopathologic examination determined presence of an adrenocortical carcinoma (pT2N0M0 ENSAT stadium II; ki67 10–15%). There was no evidence for residual or metastatic disease but given the high risk of recurrence, adjuvant therapy with mitotane was initiated. During TDM, a sudden and spuriously high level of mitotane was observed but without signs or symptoms of toxicity. After exploration, it was found that this high concentration was completely due to uncontrolled hypertriglyceridemia. After correction thereof, mitotane levels were again in the therapeutic range. This observation underscores the importance of TDM sampling in a fasting state with concurrent control of prevalent or incident dyslipidemia.

**Learning points:**

- TDM of mitotane is advocated to achieve therapeutic levels while avoiding toxicity. For correct TDM, sampling should be done at least 12 h after last intake of mitotane.
- Although sampling in fasting conditions in not explicitly mentioned in the guidelines, fasting state should be considered as elevated serum triglyceride levels might cause spuriously high mitotane levels.
- In patients undergoing treatment with mitotane and presenting with too high or unexplained fluctuating mitotane levels without signs or symptoms of toxicity, hypertriglyceridemia as a possible cause should be investigated.
- If dyslipidemia occurs in patients under mitotane treatment, other causes than mitotane (e.g. alcohol abuse and diabetes) should be considered and appropriate treatment should be initiated.

## Background

Adrenocortical carcinoma (ACC) is a rare endocrine malignancy (incidence 1 per million per year) with limited options for treatment. Prevalence is slightly higher in males than females. The illness occurs in all age groups, average age at diagnosis is 45 years. Disease etiology is unknown. Most cases are incidental diagnoses of a disease in an advanced stage with metastases already present. Approximately half of all patients suffer from excessive hormone production, often glucocorticoids or androgens. Production of estrogens by the tumor occurs less frequent, while mineralocorticoids are seldom produced. Adrenocortical carcinoma can thus present with a variety of symptoms, depending on the type of hormone produced, through mass effects or incidentally. Next to hormone-dependent symptoms, the most prevalent symptom is stomachache, and the most common clinical finding is a palpable abdominal mass. Other general symptoms include weight loss, fever, anorexia, nausea, weakness, and fatigue (1).

Chances of recovery from ACC are highly dependent on the presence of metastatic disease and whether the carcinoma can be completely surgically removed. Because these tumors are often discovered late, there is a significant chance of local and distant metastases, which results in a poor prognosis. The most prevalent locations of distant metastases are the liver (48–85%), lungs (30–60%), lymph nodes (7–20%), and bones (7–13%). Patients with residual disease after surgery or with a high risk of recurrence can be treated with mitotane. In case of very advanced disease, treatment may consist of a combination of mitotane and chemotherapy.

Mitotane is a lipophilic compound and is currently the only drug approved by both the American FDA and the European Medicines Agency for treatment of ACC. It was developed as an orally administered treatment and absorption is improved when ingested together with fatty food. Bioavailability of mitotane is approximately 35–40%, it has a high distribution volume, and the primary storage site is adipose tissue. Elimination half-life for mitotane varies between 18 to 159 days with a median of 53 days. As mitotane is a potent inducer of CYP3A4, evaluation of pharmacokinetic interactions with other drugs is advised. Therapeutic levels of mitotane will cause hypocortisolism and replacement with exogenous glucocorticoids is advocated. Also, caution for failure of the zona glomerulosa (causing hypotension and electrolyte disorders) must be exercised with possible need for fludrocortisone. Known general side effects of mitotane are nausea and anorexia, fatigue and drowsiness, headache, dizziness, myalgia and arthralgia, rash, diarrhea, hypercholesterolemia, hypogonadism and gynecomastia in men, ataxia, thyroid dysfunction, liver dysfunction, leukopenia, and others which need periodic monitoring.

Efficacy and toxicity of mitotane are related to plasma concentration. To maintain efficacy while preventing increased toxicity, the plasma concentration of mitotane needs to be between 14 and 20 mg/L, which requires therapeutic drug monitoring (TDM). Too high values are toxic and can lead to gastrointestinal and especially neurological adverse effects. Correct TDM in patients treated with mitotane requires blood samples to be acquired at least 12 h after the last dose, but it is not explicitly mentioned whether this should be under fasting conditions (2). This case presentation highlights the importance of controlling preanalytical factors in correctly performing TDM of mitotane treatment in a 38-year-old man with a non-functioning adrenal carcinoma.

## Case presentation

A 38-year-old male with a medical history of mood disorders, dyslipidemia (especially hypertriglyceridemia) and ablation for atrial fibrillation was referred to the endocrinology outpatient clinic by his general practitioner, who suspected pheochromocytoma given a recent finding of an adrenal mass on abdominal imaging. There was no family history of malignancies. Current medications were bupropion, bisoprolol, and fenofibrate. Complaints were anxiety attacks at night, together with episodic sweating and palpitations. The patient reported often feeling anxious while experiencing chest pressure and palpitations before falling asleep. Excessive sweating also occurred during the day. Additionally, the patient reported frontal headaches when looking into a light source with a particular difficulty looking outside. Over the past year, he intentionally lost a lot of weight (20 kg). There were no complaints of nausea. Virilization was normal, neither acne nor new striae were present. He did report having issues with low blood pressure from a few weeks prior, prompting the general practitioner to halve the dose bisoprolol.

## Investigation

MRI showed a nodular adrenal mass on the left with a maximum diameter of 5.8 cm and heterogenous contrast uptake, there was no loss of signal on the out-of-phase series. Two 24-h urinary metanephrine collections were normal. Late night salivary cortisol concentration was normal, as was a serum adrenal steroid hormone profile except for mildly elevated 17-OH-progesterone (2.68 ng/mL; reference values <1.27 ng/mL).

### Treatment

Considering the size of the lesion and the inability to exclude ACC, surgical resection was advised. In September 2020, a left-sided laparoscopic adrenalectomy was performed. The tumor was completely removed, and no metastases were suspected. Histopathological examination showed a morphological and immunohistochemical image to be considered as adrenocortical carcinoma (pT2N0M0 ENSAT stadium II; Weiss score of 5, ki67 10–15%). In consideration of a fairly high risk of recurrence, adjuvant treatment with mitotane was started for a period of 2 years minimum. The oncological follow-up consists of periodical FDG-PET/CT scans (every 4–6 months initially) combined with evaluating steroid hormone profiles.

The patient started taking mitotane 500 mg (2 co/day) in late September 2020 and increased doses weekly until 3 × 3 co/day after 3 weeks. As mitotane is a strong corticolytic and adrenolytic drug, it can cause suppression of endogenous cortisol production. In addition, mitotane strongly induces production of cortisol binding globulin as well as the activity of several steroid-metabolizing enzymes. As such, the patient was also administered hydrocortisone 3 times per day (hydrocortisone acetate 20–10–10 mg). Guidelines concerning the necessity of applying hydrocortisone stress instructions were provided to the patient. Tolerance for mitotane was relatively good, without severe nausea or diarrhea. There were minor complaints of flatulence and ructus. The patient felt quickly irritated and complained of fatigue. Additionally, he reported occasional dizziness when standing up and had recently tested positive on a tilt test (fainted). Biochemically, only a mild leukopenia was observed.

### Outcome and follow-up

**Figure 1:**
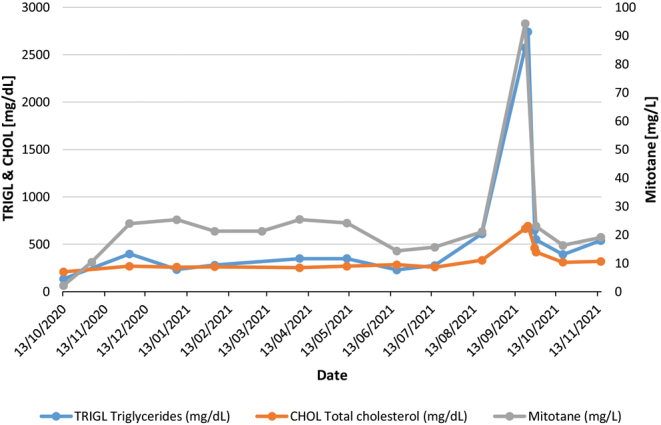
Evolution of triglyceride, total cholesterol, and mitotane levels during treatment.

After initiation of mitotane, TDM and monitoring of side effects and toxicity was started (Fig. 1). Mitotane levels slowly but steadily increased until early December 2020, reaching plasma levels of 24 mg/L. The patient suffered increased mood swings and intermittent nausea, which improved after taking 10 mg hydrocortisone extra. Mitotane was stopped for a few days and restarted at 3 times 1000 mg daily as soon as these symptoms disappeared. Hydrocortisone dosage remained unchanged. Upon the next TDM in January 2021, the mitotane plasma level was 25.3 mg/L but without any complaints from the patient. Biochemically, the known leukopenia remained stable, and an FDG-PET/CT indicated no arguments for recurrence or distant metastases. The mitotane dosage was decreased to 5 × 500 mg daily.

The following three months, no complaints out of the ordinary were reported, prompting mitotane dosage to be maintained until April 2021. Around then the patient reported a resurgence of nightly episodes of palpitations and sweating. He suspected hydrocortisone overdose although actual total daily dosage was 40 mg with the last intake in the late afternoon. He also reported a pesky testicular feeling, not real pain. An ultrasound of the scrotum was reassuring. Mitotane plasma levels were 25.4 mg/L; therefore, mitotane dose reduction to 3 × 500 mg and 4 × 500 mg every other day was advised.

In May 2021, the mitotane plasma level remained elevated at 24.14 mg/L. Other blood tests were normal, except for slightly raised gamma-glutamyltransferase and lower free testosterone levels. The patient did mention significant physical deterioration, having a decreased libido since a couple months prior, and having painful nipples when lying down. Mitotane dosage was further reduced to 2 and 3 tablets every other day. In the next 2 months, mitotane levels were on target and the patient did not report specific symptoms. Mitotane dosages was modestly increased to 3 × 500 mg per day in June, and because of persisting hypercholesterolemia, treatment with simvastatin (20 mg/day) was started.

In August 2021, mitotane plasma levels had increased to 21 mg/L and cholesterol levels were markedly increased. The patient experienced fluctuating blood pressures during the day as well as a continuous sensation of thoracic pressure, a feeling of arrhythmic heart beats and an oppressive sensation in the throat which improved after increasing hydrocortisone dosage. The patient did not tolerate simvastatin well and this treatment was stopped, mitotane dosage remained unchanged. Upon TDM in September, however, mitotane levels suddenly increased to 94.3 mg/L. Remarkably, there were no clear signs or symptoms of mitotane toxicity. Upon questioning, the patient confirmed that the sample was drawn while fasting and before intake of mitotane and that no new drugs were started but did report that he took his total daily dose of mitotane (3 × 500 mg) at once the evening before. To account for laboratory abnormalities or assay interference, the mitotane analysis was repeated in three different runs and similar results were obtained. Additional biochemical analyses showed the known mild leukopenia but did reveal an extreme hypertriglyceridemia (2600 mg/dL). Apparently, the patient had been without fenofibrate for 1 week and he also admitted having significantly exceeded his dietary restrictions during the previous days. Mitotane was stopped immediately, while fenofibrate and dietary measures were reinstated. During the following days, the patient complained of nonpersistent nausea and known concentration disorder. There was no vertigo. One week after stopping mitotane intake, triglycerides decreased to 650 mg/dL and mitotane plasma level was 23.6 mg/L, while other blood tests remained normal. Mitotane was restarted at a dosage of 2 × 500 mg daily.

During further follow-up until the end of mitotane treatment, mitotane levels remained largely within the therapeutic window and there were no iconographic nor biochemical arguments for recurrent disease.

## Discussion

The extremely high mitotane plasma levels in our patient are distinctly associated with high levels of triglycerides. Two possible mechanisms could explain this. On the one hand, mitotane association with lipoproteins has been reported (3) while on the other hand, mitotane can lead to an increase in serum lipid levels (4).

Regarding the first mechanism, it is important to remember that mitotane is a very lipophilic drug and circulates in the bloodstream bound to plasma lipoproteins (HDL, LDL, and VLDL). In case of hyperlipemic serum, the very high levels of some lipoproteins – VLDL in particular – can increase the amount of circulating mitotane in plasma. Kroiss *et al.* (5) confirmed that the majority of circulating mitotane is bound to lipoproteins and investigated the impact of lipid composition on mitotane bonds with individual lipoproteins. They found that mitotane content in the different lipoproteins showed a positive correlation with both cholesterol and triglyceride content, that sum value of triglyceride and cholesterol content correlated best with plasma mitotane levels, and that the relative abundance of mitotane in cholesterol- and triglyceride-rich VLDL particles was higher in patients with higher total cholesterol and triglyceride serum concentrations. Also, the authors confirmed that lipoprotein free mitotane is the active form of the drug as lipoproteins bonding impedes the bioavailability of mitotane. Finally, according to the authors, clinical experience has shown that supratherapeutic mitotane levels due to hyperlipidemia are not accompanied by significant symptoms, as was the case with our patient.

Importantly and somewhat confusing, however, patients can develop severe hypertriglyceridemia during mitotane treatment. Indeed, mitotane can induce or aggravate preexisting hypercholesterolemia or hypertriglyceridemia through HMG-CoA-reductase activation and a retrospective analysis on a large patient cohort indicated an increase of HDL-cholesterol levels of approximately 60% independent from treatment with lipid-lowering drugs (6).

To conclude, this case report illustrates the importance of controlling confounding factors when performing mitotane TDM. Especially in the case of (extremely) supratherapeutic mitotane levels without associated signs or symptoms, hyperlipidemia as an explanatory condition should be ruled out to avoid unnecessary dose reductions.

## Declaration of interest

The authors declare that there is no conflict of interest that could be perceived as prejudicing the impartiality of the study reported.

## Funding

This research did not receive any specific grant from any funding agency in the public, commercial, or not-for-profit sector.

## Patient consent

Written informed consent for publication of their clinical details and/or clinical images was obtained from the patient/parent/guardian/relative of the patient
